# Node Immunization with Time-Sensitive Restrictions

**DOI:** 10.3390/s16122141

**Published:** 2016-12-15

**Authors:** Wen Cui, Xiaoqing Gong, Chen Liu, Dan Xu, Xiaojiang Chen, Dingyi Fang, Shaojie Tang, Fan Wu, Guihai Chen

**Affiliations:** 1 School of Information and Technology, Northwest University, Xi’an 710127, China; cuinwu@gmail.com (W.C.); gxq@nwu.edu.cn (X.G.); liuchen@nwu.edu.cn (C.L.); xudan@nwu.edu.cn (D.X.); xjchen@nwu.edu.cn (X.C.); 2 Naveen Jindal School of Management, University of Texas at Dallas, Richardson, TX 75080, USA; 3 Department of Computer Science and Engineering, Shanghai Jiao Tong University, Shanghai 200240, China; fwu@cs.sjtu.edu.cn (F.W.); gchen@cs.sjtu.edu.cn (G.C.)

**Keywords:** node immunization, strategy, social network

## Abstract

When we encounter a malicious rumor or an infectious disease outbreak, immunizing *k* nodes of the relevant network with limited resources is always treated as an extremely effective method. The key challenge is how we can insulate limited nodes to minimize the propagation of those contagious things. In previous works, the best *k* immunised nodes are selected by learning the initial status of nodes and their strategies even if there is no feedback in the propagation process, which eventually leads to ineffective performance of their solutions. In this paper, we design a novel vaccines placement strategy for protecting much more healthy nodes from being infected by infectious nodes. The main idea of our solution is that we are not only utilizing the status of changing nodes as auxiliary knowledge to adjust our scheme, but also comparing the performance of vaccines in various transmission slots. Thus, our solution has a better chance to get more benefit from these limited vaccines. Extensive experiments have been conducted on several real-world data sets and the results have shown that our algorithm has a better performance than previous works.

## 1. Introduction

To what extent have you been bothered by the outbreak of undesirable things, a contagious disease, computer virus, or malicious rumor, and gone through a difficult time to wait for the constraint of the outbreak? Minimizing the spread of undesirable things is a major problem in Centers for Disease Control, Kaspersky, Twitter and Facebook, etc., where management and staff undertake much more responsibility for discovering and controlling the contamination. Many organizations are looking to address this problem with the distribution of vaccines/patches in the relevant network with a limited budget [1,2,3]. For example, before the outbreak of H1N1 Flu, American Centers for Disease Control and Prevention(CDC) had centralized the distribution of vaccines for nearly 150,000 sites (hospitals, clinics, health departments), and it had been based on a cautious strategy of distributing the limited budget of vaccines to protect more people from being infected. Current placement strategies of vaccines/patches, however, are assuming that the initially infected nodes are randomly appearing [4,5,6,7]. Almost all cases, however, are such that the initially infected nodes are unevenly distributed. For example, in a city, some susceptible individuals are at high risk of getting an infection from a particular infectious disease (e.g., poultry farmers who are easily exposed to bird flu). Similarly, once a computer virus was made, it is aiming at the servers which lack specific precautions rather than randomly attack servers, and the propagation of this virus will start from those servers, obviously. Knowing the initially infected nodes as a prior information is also indispensable for the rumor control of social network companies. They could send an official alert message to limited people to prevent the rumor from developing into a public affair. With the help of a reliable node protection strategy, the related social network applications also can be extended to other hot topics, such as minimizing the error message propagation in Cyber-Physical Systems [8,9,10,11,12,13,14], which already attracts a lot of attention from the academic field and industry areas [15,16,17,18,19]. Besides, the dissemination of data in a network [20,21,22,23,24] can also be guaranteed in a better way.

Minimizing the influence of undesirable things under prior information has been recently proposed [25,26] and the key part of solving this problem is to propose a greedy vaccines placement algorithm. Researchers try to improve protection performance of their algorithm by modifying heuristic idea since the problem is **NP-hard** [27] and even computing the influence between each node is a **#p** problem [28]. However, underlying their solutions is an assumption that all vaccines need to be deployed at once, which does not relate well to the facts. The saving number of one vaccine may have a notable difference at a different time with various network structures. Thus, the immunization strategy should take the benefit of each vaccine at different time slot into consideration so that we can maximize the utilization of every vaccine. This paper introduces Dancing with the propagation virus (DWPV), a dynamic vaccines placement strategy combines the time division into our solution. In line with common practice in selecting vaccines set [25,26,29,30], DWPV adopts Independent Cascade (IC) model [27] as virus propagation model. To get the placement position of vaccines, the propagation model is a basic function to analyze the influence of immunizing those candidate nodes. The challenge of our problem, however, is to identify where and when the vaccines should be distributed.

**Figure 1:**
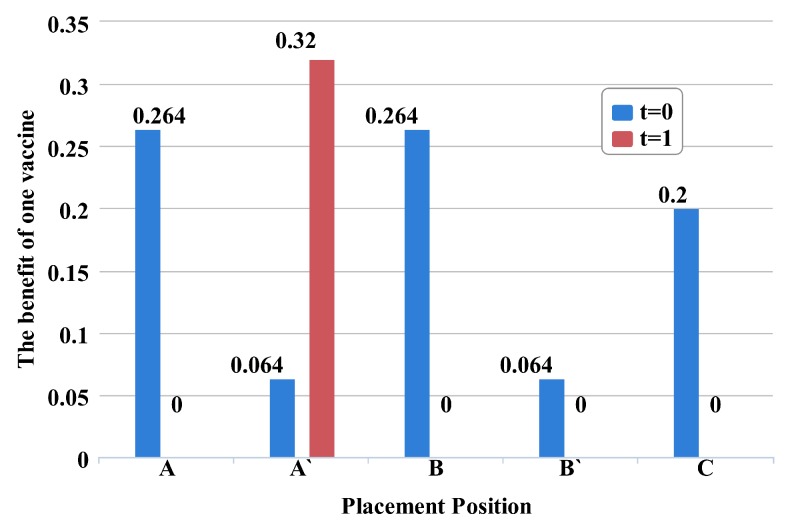
Different benefit of position over each placement time.

Unlike past approaches [6,7], which consider delaying the placement time as detrimental, DWPV exploits the postponement of vaccine distribution to protect healthy nodes from being infected. Specifically, the benefit of putting one vaccine in a specific position on the network is always changed by the dynamic network structure. Hence, if we can ensure that a higher benefit of vaccines can be obtained from other transmission time slot (as shown in Figure 1), then we postpone the placement time of those vaccines to next time slot to save more nodes.

**Figure 2:**
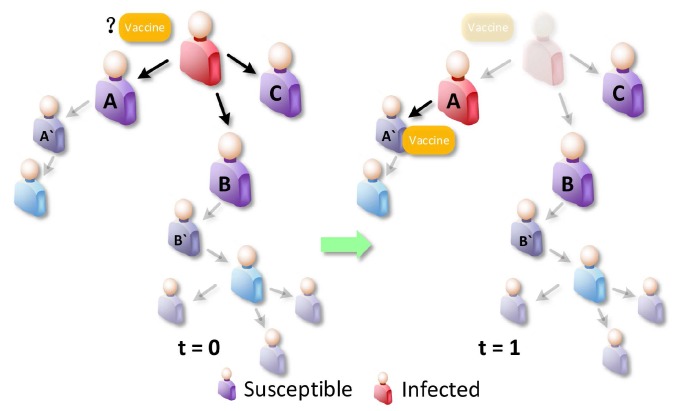
Intuition underlying DWPV use of time division in distributing vaccines: The figure shows an infected node (in red) and some susceptible nodes (in blue). The infected node has one chance to infect their neighbors at *t* = 0. At *t* = 1, the node A has been infected by the initially infected node and other nodes (B and C) are lucky ones.

To illustrate DWPV’s approach, Figure 2 shows a toy example with some susceptible nodes and one infected node, where the infected node is surrounded by three neighbors A, B and C, and the virus propagation probability is 0.2. As the figure shows, the idle vaccine, like the yellow one, may have a very limited benefit from the neighbors of the infected node at time slot 1 as we are not really sure who will be infected in the next time slot. However, this vaccine tends to have significantly different benefit from being distributed at time slot 2, e.g., putting the vaccine on A’. Hence, one needs to consider the full-time series benefits of vaccines; one-time slot alone, like the benefit from time slot 1, can be invalidated. To see this more clearly, we can use the standard metric [25] to measure the effectiveness of putting vaccine on the each node of Figure 2. Figure 1 shows the obtained benefit from each node of Figure 2 at t = 0 and t = 1. It is clear from this figure that looking at the neighbors of initially infected alone—i.e., A, B and C at t = 0—would wrongly indicate that vaccinating node B is the best choice, while looking at t = 1 allows us to realize that vaccinating node A’ at t = 1 has more benefit than vaccinating node B at t = 0. Thus, a robust vaccine placement strategy needs to compare the node’s benefit at different time slots by quantifying how much difference there is between putting vaccines on the current time slot and next time slot.

So how can we automatically quantify the changes across the benefit of different time slots? To do so, we need to estimate the influence of nodes’ benefit in different time slots. In contrast to the illustrative example, however, real-world networks always have many potential propagation events, which may change differently depending on the number of initially infected nodes and the propagation probability of each node. Further, different scale of networks typically has a different complexity of relationship between each node, causing the benefit of nodes to be changed frequently. In designing a technique that finds the most suitable node and its placement time despite these changes, we are inspired by the baseline strategy [25], which without consideration the placement time of vaccines, in using the dominator tree to approximately represent the relationship between each node of the network. Given an arbitrary graph and trying to figure out the relation between each node is a **#p** problem to us, we need to approximately represent the dominating relation to finding the dominator node that we can put a vaccine on there to release the most infecting pressure of other nodes. In graph theory, a graph is converted to a dominator tree following the rules that if the status of A is donated by B then we define it as B denote A. Namely, we can directly point out the dominating node after we translate the original graph into a tree structure and merge the initially infected nodes as the root node. As opposed to only converting the original graph into the dominator tree and point out the key node, DWPV analyzes the transmission probability of each root node’s neighbor, which nodes are treated as the best candidate nodes in previous works, by using a binary tree to figure out the expected benefit of these nodes in current time slot and next time slot. Furthermore, we propose a Monte Carlo sampling method as a substitute method to suit the limited computation application. We present the design in Section 4 and we have theoretically proved that the strategy of ours, which combine the time division into the placement of vaccine, has an advantage over previous works in controlling the propagation of the virus.

Finally, while we could obtain a set of position to put the vaccines and its corresponding placement time, there will always be many changes in the propagation process of the virus, since all analysis results are based on a probabilistic model. For this reason, DWPV decides to put one vaccine on the graph only if a neighbor of the candidate node is infected. This design broadens the assurance of the effectiveness of vaccines (up to 30%), and in case of some vaccines, they become invalid as their neighbors are always safe during the propagation of the virus.

This paper makes the following contributions:

1. It presents a vaccine placement strategy that exploits the changes of vaccine’s benefit in different time slots to get the highest benefit of one vaccine. As a result, the design delivers high-level protection to keep more healthy nodes from being infected.
2. It also demonstrates the capability of the dominator tree to select out the candidate immunization nodes in a dynamic scenario, and we successfully use it to protect more healthy nodes in an infected network than the static one. While our design and results are presented in the social network, the basic idea can be extended to other communication problems.
3. It presents an accurate method to evaluate the possible benefit from other time slots by using binary tree and its simplified version with lower computation complex by using Monte Carlo method.
4. It has been evaluated in some real-world large-scale data sets.

The rest of the paper is organized as follows. Section 2 introduces our preliminaries. Section 3 define our problem as vaccines placement problem and we present our solution in Section 4. Section 5 shows our experiment results with open-resource datasets. Section 6 is the related work about this paper and we have a conclusion of this paper in Section 7.

## 2. Preliminaries

The information propagation model is the first thing we have to understand for knowing the method of restricting the malicious things communication in the social network, which is depicting the propagation process of information (bad/good) in a discrete way. The Susceptible/Infected/Recovered (SIR) Model [1] and Independent cascade (IC) model [27] are two popular models about malicious information communication which have been well studied for quite a long time. Compare to the SHIR (Susceptible-Hidden-Infected-Recovered), SIS(Susceptible-Infected-Susceptible) and LT (Linear Threshold), those two models are more widely used. In this paper, we introduce our method in IC model first, and we transfer the IC model into SIR model later. Meanwhile, notations list is shown in Table 1.

**Figure 3:**
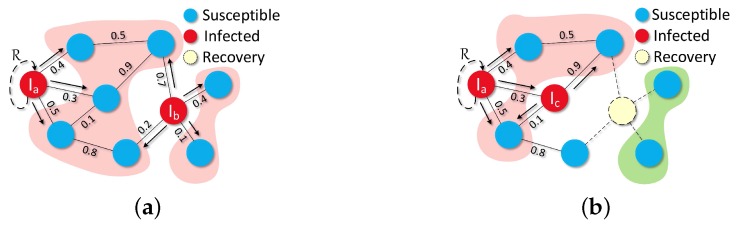
SIR transmission process: At t=0, $I_{a}$ and $I_{b}$ are the initial infected nodes in G(V,E). Those two nodes are trying to infect their neighbors through weighted connection edges. At the same time, both $I_{a}$ and $I_{b}$ are struggling to become recovered by using their own antibodies, the probability of occurrence of this event is *R*. At t=1, a new infectious node $I_{c}$ is coming and trying to infect its neighbors, fortunately, $I_{b}$ overcame the hateful disease and protected its neighbors (which are in the green area) from being infected. (**a**) t=0; (**b**) t=1.

SIR Model is a comprehensive description model of disease spreading, which has been well studied for many decades. As shown in Figure 3, initial infected nodes $I_{0}$ are already in the graph G(V,E). Besides, the weight of each edge, P(u,v) (0<P(u,v)<1), is used to represent the *transmission probability* between *u* and *v*. Here, if one node gets infected at step *t*, then that node will try to infect its neighbors (*susceptible*) in G(V,E) at step t+1. The status of each node will be updated in every time slot and it is notable that the recovered nodes will be removed from G(V,E). It is not until there is no infectious node in the G(V,E) that the transmission processing of virus is over. Besides, once a node gets infected, that node will try to transform its status from *infected* to *recovery* (something like a man has antibodies) and the probability of this transformation is determined by R.

In general, in order to concentrate on the propagation process of the undesirable thing, we always use IC model to instead of SIR model [31,32,33]. Compared to SIR model, IC model omits the process of recovery (R=1) and every infected node has only one chance to infect its neighbors. In this paper, we adopt IC model as our basic model too, and we will introduce how to transform IC to SIR in Section 4.

## 3. Problem Definition

The influence minimization problem is like the *Vaccines Placement* (VP) problem, which is focusing on restricting the influence of malicious information in the social network. Based on the knowledge of information propagation model, we will give a formal definition of *Vaccines Placement* (VP) problem for knowing its optimization objective and problem complexity to specify a suitable method for our problem.

### 3.1. Vaccines Placement Problem

We are aiming at getting the maximum number of healthy nodes in the network, which means that we need the higher value of $\varphi_{G,I_{0}}\left(V\right)$ at t=T ($I_{T}=\emptyset ,t\in \left(0,T\right)$) in a weighted graph G(V,E) with initial infectious nodes set $I_{0}$ and IC based propagation model. Namely, the target is to solve the *Vaccines Placement* (VP) problem that is to find the appropriate vaccines set V so that we can save the maximum number of nodes, where V⊂H⊂V and H is the subset of *V* represent healthy nodes in G(V,E). Formally,

(1) $$\begin{array}{c} \max\;\;\varphi_{G,I_{0}}\left(V\right)\\ s.t.\;|V|=k. \end{array}$$

Once we have the formal description of our problem and the simple example of Figure 2, we could know that only knowing the position of vaccines is not enough for us to get the richest effectiveness of the limited vaccines. We should have taken the time division of immunization into our strategy to ensure the effectiveness. For this reason, we separate the original VP problem into two individual problems, a static one and dynamic one, to emphasize the importance of the immunization time and eventually get more healthy nodes in the network.

#### 3.1.1. Static Vaccines Placement Problem

After being carefully selected from H, naively, ∀vaccine⊂V needs to be put on G(V,E) at t=0, as if we cannot wait any longer. Then, we define this kind of problem which selects V only at t=0 as SVP problem, since V is aiming at reducing the damage just caused by $I_{0}$ which has less consideration of the changes about $I_{1}\to I_{T}$.

#### 3.1.2. Dynamic Vaccines Placement Problem

The main difference between DVP$\left(G,I_{i},t\right)$ and SVP$\left(G,I_{0}\right)$ is that DVP$\left(G,I_{i},t\right)$ has a *time dimension t*. As the IC model is a stochastic model, we consider that the V may be not fixed. Specially, we take the deploying time of vaccines into consideration to save healthy nodes, where i⊂(0,T), and we believe that V can be adjusted for $\max\{\varphi_{G,I_{t}}\left(V\right)||V|=k^{*},V\subset H\}$ at different *t*, where $k^{*}$ represents the changing value of *k* since some vaccines deployed at different *t*. Therefore, based on the above points of view, we can get the Remark 1 as follows.

**Remark** **1.** *SVP problem is a special case of DVP problem, SVP* ⊂ *DVP*.

### 3.2. Complexity of Vaccines Placement Problem

Unfortunately, the VP problem is **NP-hard** [27]. More over, the greedy algorithm cannot be easily used as the best approximated solution [25], since $\varphi_{G,I_{0}}\left(V\right)$ is not a submodular function, which means VP problem do not satisfied with the law of diminishing marginal utility that f(V∪{v})−f(V)≱f(U∪{v})−f(U), where ∀v⊂V and V⊂U⊂H.

**Theorem** **1.** Dynamic Vaccines Placement Problem is **NP-hard**

**Proof.**  As mentioned in Remark 1 that SVP is a special case of DVP, and after adding the *time dimension* into SVP, the complexity of DVP is increased rather than be reduced. Namely, every selecting scheme has the same computation level as SVP has, and we need to perform the selecting algorithm multiple times at each time slot until k* from *k* to 0. Furthermore, in [25], the SVP problem has been reduced from a MinKU set [34] problem which has been proved as an **NP-hard** problem. Thus, the DVP problem is not **NP-hard** either. ☐

From [28] we can ensure that the infection probability between two nodes cannot easily be acquired, which has been approved as **#p** problem [28]. Given any constant 0<ϵ<1/3, there exists a $m_{\epsilon }$ such that the SVP problem with $m>m_{\epsilon }$, cannot be approximated in polynomial time within an absolute error of $\frac{1}{2}m^{1-2\epsilon }+\frac{3}{8}m^{1-3\epsilon }-1$ unless P=NP. Therefore, it becomes our prime purpose that we need to design a heuristic algorithm for solving the SVP problem.

## 4. Placement Strategy of Vaccines

Immunization time of limited vaccines, as we already knew, is an important key to satisfied our nodes protecting need. However, as the network structure is difficult enough that we can not get the immunization time and position easily from the original graph. Naturally, we need some basic tools of graph theory to initialize our network so that the structure can be simplified to meet the demand of analyzing the influence of each candidate node’s position. Once we get the easy network, we could propose our heuristic idea of the VP problem and verify its advantages over previous static version.

### 4.1. Network Initialize

The VP problem has been proved to be an **NP-hard** problem in the previous section and it needs a heuristic method to be solved. As mentioned before, the essence of our problem is a graph problem. However, knowing the influence relationship between two nodes is a **#p** problem, and we need to design a method to solve our problem in polynomial time. Hence, we initialize the network to simplify our problem. In this section, we will introduce the initialize methods, like dominator tree and *super infected nodes*.

#### 4.1.1. Dominator Tree

**Figure 4:**
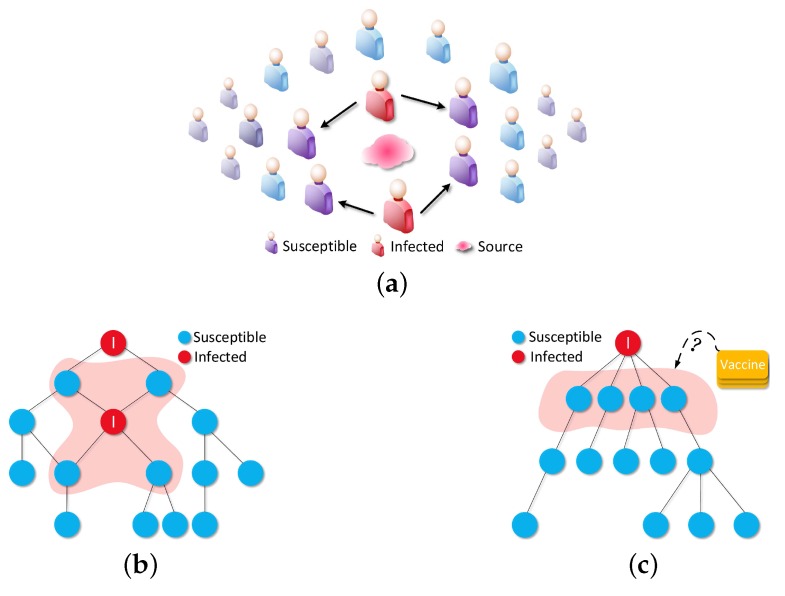
Fundamental Knowledge of Our Method: At first, some miserable people had caught a malignant virus and it would be spread to more and more people who have connection with those infected men; Then, we translated the abstract of social network into graph G(V,E) for designing an appropriate method to control the malignant virus; At last, we convert G(V,E) into DT by combing the infected nodes as one super infected node and let that super infected node to be the root of DT. Once we have DT, we just need to select out the *best-k-benefit* nodes in MDL. Finally, we can get the vaccines set V at t=0 for the SVP problem. (**a**) People infected; (**b**) G(V,E); (**c**) Dominating Tree(DT).

For solving VP problem, after being given a graph G(V,E) with initial infected nodes set $I_{0}$, the selection process of V not only need to find out the *Most Influence Node Set* (MINS) but also need to take the relationship between $I_{0}$ and MINS into consideration. Meanwhile, getting the relationship between each node is a **#p** problem, then we need an approximate method to represent the relationship. Luckily, the Dominator Tree (DT, as shown in Figure 4c that is a tree with infected root) has the power to convert a graph into tree structure with a dominating relation in linear-time, e.g., if node *u* wants to infect *v*, all the path between *u* and *v* need to pass *m*, then we define it as *m* dom *v* (or m→v). After having a tree structure to represent G(V,E), then we just need to treat those nodes, which are in the *Most Dominating Layer* (MDL) of DT (the first layer after the root node, e.g., the pink area as shown in Figure 4c, as the best candidates.

**Definition** **1** (Most Dominating Layer)**.** *After being given a DT with infected root, then we define the first layer after the root node as the Most Dominating Layer (MDL). MDL is a node set that nodes in this layer not only has the highest probability to be infected by the infectious root node of all other nodes but also can determine if their children will be infected or not. Obviously,* |*MDL*| < *k is always true, otherwise we can easily stop the propagation of virus just by removing all direct suspectable nodes.*

#### 4.1.2. Benefit of One Node

Calculating the influence of removing one node in G(V,E) is a **#p** problem problem, which has been proved by [28]. However, in DT, we can calculate out the value of influence (we define it as *benefit*) with an easy method since each branch of a tree structure is mutually independent. Thus, we can treat *benefit* as an estimation metric to estimate the value of candidate nodes so that we can make a better choice from those nodes.

**Definition** **2** (Metric for Estimating the Value of Removed Node)**.** *In DT, every node has a* benefit *which is used to represent the expected benefit of removing itself from G*(*V*, *E*)*, and that is defined as a metric for estimating the value of the removed node. As every branch in DT is independent, the calculation of the benefit is a linear time algorithm and the details has been shown in Algorithm 1.*

**Figure 5:**
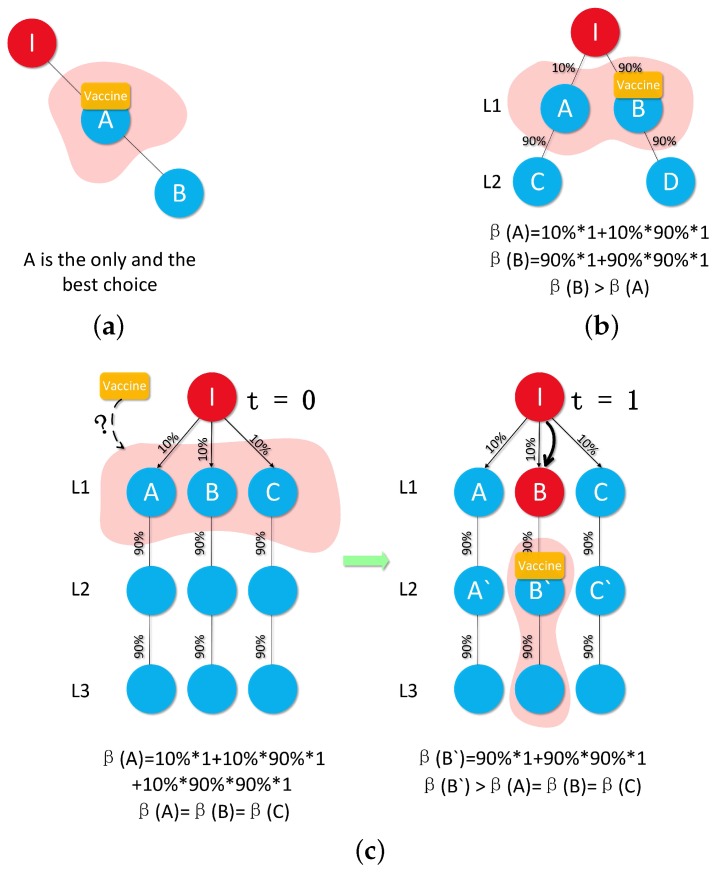
Examples for one vaccine: A simple case like *Simple Case 1* that we can put the only one vaccine on node A with no doubt. *Simple Case 2* is also not a hard decision for us. After comparing the benefit between A and B, we can put vaccine on B to obtain a bigger $V_{B}$. However, in *DVP Case*, after investigating the difference of $V_{B}$ between t=0 and t=1, surprisingly, we discovered $V_{B}^{t=0}<V_{B}^{t=1}$. That is to say, the value of $V_{B}$ is changed by time, in addition, a bigger $V_{B}$ can be obtained by another time. There is a big difference between waiting some time slots and without waiting. In previous assumption, we must put the V on G(V,E) at t=0 which means they could not get the bigger $V_{B}^{t=1}$. (**a**) Simple Case 1; (**b**) Simple Case 2; (**c**) DVP Case.

In Algorithm 1, β(i) is calculated by a recursion function. Line 3–5 depict that the calculating process will be kept until it down to the leaf node. In DT, as for each branch is independent, the complexity of this algorithm is O(n). Meanwhile, a vivid instance of Algorithm 1 is shown in Figure 5.
**Algorithm 1** Benefit of one candidate vaccine.**Input:** One candidate vaccine *i*, *P*_*u*,*v*_ and a dominator tree with infected root node *I*_0_**Output:** The benefit of node *i* and it is stated as *β*(*i*)**Function** Cal-Benefit (node *i*)1:**if** *i* is not a leaf node **then**2:  β(i)=13:  **for** each child *j* of node *i* **do**4:      $\beta \left(i\right)=\beta \left(i\right)+\mathrm{Cal}-\mathrm{Benefit}\left(j\right)*P_{ij}$5:  **end for**6:**else**7:  β(i)=18:**end if**9:**return** β(i)**EndFunction**


Then, we can transform our target function (Equation (1)) into

(2) $$\begin{array}{c} \max\;\;\phi_{DT,I_{0}}\left(V\right)\\ s.t.\;|V|=k. \end{array}$$

Here, we use the calculation of the maximum summation of expected benefit $V_{B}$ to stands the selection of V. Moreover, as $V_{B}\propto \max\;\;\phi_{DT,I_{0}}\left(V\right)$, we can constrain our target into calculating the value of $V_{B}$,

(3) $$V_{B}=\max\sum_{j=1}^{j=k}\beta_{j}\left(i\right),i\subseteq V.$$

### 4.2. The Heuristic Idea of VP Problem

From the point of adding time division into our solution, heuristically, we should compare the advantage of vaccines in each time slots and decide which slots is the best immunization time finally. Moreover, we discover that $V_{B}$ can be obtained from other layers of DT by waiting for some time slots, rather than just from the MDL.

**Lemma** **1.** *Given a DT with infected root node, we should not just treat the nodes in MDL as optimal candidates for Equation* (2) *since sometimes we can own a bigger* $V_{B}$ *from other layers of DT by waiting some transmission time slots.*

**Proof.**  For now, we are using Figure 5 as an example to prove our view and we assume the number of vaccines is one to ease of calculation. (1) We can simply vaccinate the node *A* in Figure 5a; (2) In Figure 5b, by using Algorithm 1, we can calculate out β(A)=0.19 which means we can expectedly save 0.19 healthy node. At the same time, β(B)=1.71 which is bigger than β(A). Then, we decide to put vaccine on *B* for a bigger $V_{B}$ so that we can save more nodes; (3) As shown in Figure 5c, if we are using the strategy of SVP, then we will save 0.271 max. Whereas, we are pondering whether we can get a bigger $V_{B}$ by adding the *time division* into our strategy. That is, we wait for one time slot and do nothing with the MDL (L1, as shown in Figure 5c), then $G_{I_{0}}^{t=0}\left(V,E\right)\to G_{I_{1}}^{t=1}\left(V,E\right)$ and $I_{0}\to I_{1}$. At that time, if some nodes get infected at t=1, then we can surely obtain a bigger $V_{B}$ in L2 as for the benefit of each node in L2 is much bigger than in L1. Otherwise, no one gets infected which means that we do not even use vaccines, but this is a small probability event. ☐

### 4.3. Prove the Advantages of DVP$\left(G,I_{i},t\right)$ over SVP$\left(G,I_{0}\right)$

After G(V,E) is converted to DT, obviously, we can get β(i)>∀β(j) so long as without taking *time dimension* into consideration, where i⊂MDL, j⊂C(i), C(i) is the children set of node *i*. However, as lemma 1 shows, once we aware that β(i) will not always bigger than β(j), where j⊂C(i), then the *time dimension* becomes an indispensable factor of VP problem. Namely, we need to take *time dimension* into our strategy to determine whether we should put vaccines at t=θ or t=i, where i⊂(1,T), i>θ, *θ* represent the current moment. So, we need to cautiously compare the expected benefit of V at t=θ and t=θ+1, until $k^{*}=0$ or t=T. At the same time, if we can own some bigger *β* at t=θ+1, then we can wait to t=θ+1 to obtain a bigger $\varphi_{G,I_{0}}\left(V_{t=\theta +1}\right)$. For better understanding, we need to look back to Figure 5c. At t=0, we can have 0.271 of benefit max, as we only put vaccines on L1. Whereas, at t=1, as have already known that node *B* is infected, then we can get a bigger $\varphi_{G,I_{0}}\left(V_{t=1}\right)$ as 1.71.

**Lemma** **2.** *The benefit of each node is time-sensitive. Namely, in each propagation round, the structure of* G(V,E) *will be changed by infected nodes and these changes directly impact the benefits.*

**Proof.**  As for the transmission model we based on, the infected nodes set will keep developing until no one is infected in G(V,E). Once one node gets recovered, it will be removed from the network and the G(V,E) will not be changed either. Sometimes these changes will bring a bigger value of the benefit of V as compared to previous time slot, and sometimes they bring a smaller one. Meanwhile, β(i)>β(j) means that immunizing *i* can save more healthy nodes than immunizing *j* on average. Thus, as $I_{\theta }\to I_{\theta +1}$, the value of $\varphi_{G,I_{0}}\left(V\right)$ must be changed by adjusting the placement time of vaccine, sometimes bigger and sometimes smaller. ☐

**Theorem** **2.** *The strategy of obtaining the maximum value of* $\varphi_{G,I_{0}}\left(V\right)$ *is related to every time slot* t⊂(0,T)*, rather than just about* t=0 *as previous works assumed. Moreover, the strategy of DVP*$\left(G,I_{i},t\right)$ *will get more benefit* $\varphi_{G,I_{0}}\left(V\right)$ *than SVP*$\left(G,I_{0}\right)$ *does, as*

(4) $$\varphi_{G,I_{0}}^{DVP(G,I_{i},t)}\left(V\right)\geq \varphi_{G,I_{0}}^{SVP(G,I_{0})}\left(V\right).$$

**Proof.**  Shortly, combining the idea of lemmas 1 and 2, we know that the strategy of DVP$\left(G,I_{i},t\right)$ can save more healthy nodes. Specially, the vaccines set V of DVP is selected from multi time slots, only when the benefit of some candidates in $V_{t=\theta +1}$ is bigger than in $V_{t=\theta }$ can these candidates of V wait to t=θ+1. Besides, the worst case of DVP$\left(G,I_{i},t\right)$ is that there is no node has bigger benefit as compared with SVP$\left(G,I_{0}\right)$. At that time, DVP$\left(G,I_{i},t\right)$ and SVP$\left(G,I_{0}\right)$ will have the same V. Thus, $\varphi_{G,I_{0}}^{DVP(G,I_{i},t)}\left(V\right)\geq \varphi_{G,I_{0}}^{SVP(G,I_{0})}\left(V\right)$ is valid. ☐

### 4.4. Immunization Time Comparing

As mentioned previously, the core of our approach not only selecting out the right nodes to put vaccines on it but also choosing the right time to put it on G(V,E). To do so, we need to find out that if there have $\beta {\left(i\right)}^{t=1}>\beta {\left(j\right)}^{t=0}$, where $i\subset MDL^{t=1}$ and $j\subset MDL^{t=0}$. Then, we put those nodes that has bigger *β* at t=0 and keep others idling until t=1. The computation is a cyclic operation till the $k^{*}=0$, then we will get the $V_{B}$ as announced at Equation (3).

The details of *Find the most valuable set of vaccines* is shown in Algorithm 2. After knowing the $I_{\theta }$ then we calculate out the $V_{I_{\theta +1}}$, and accurately predict the $I_{\theta +1}$ then we calculate out the $V_{I_{\theta +1}}$, in addition, both sets of vaccines are sorted by *β*. Then we can let these node wait to t=θ+1 to obtain a higher $\varphi_{G,I_{0}}\left(V\right)$.
**Algorithm 2** Find the most valuable set of vaccines.**Input:**
G(V,E), *k*, $P_{u,v}$, infected set $I_{0}$ and $I_{1}$, most-k-benefit vaccines set $V_{I_{0}}$ and $V_{I_{1}}$**Output:** Vaccines set V
1:$k^{*}=k$2:**while** $I_{1}\neq NULL$ and k>0 **do**3:  **for** i=0 **to** *k* **do**4:      j=05:      **if** $V_{I_{0}}\left[i\right]\geq V_{I_{1}}\left[i\right]$ **then**6:            Updating the V[i] by $V_{I_{0}}\left[i\right]$7:            j=j+18:      **else**9:            Updating the V[i] by $V_{I_{1}}\left[i\right]$10:      **end if**11:  **end for**12:  k=k−j13:  $I_{0}\leftarrow I_{1}$14:  $I_{1}\leftarrow I_{2}$15:**end while**


As shown in Algorithm 2, it is easy to see that to get the V at a different time is also a major component of DVP strategy. So, we need to focus on the method of calculating those $V_{t=i}$, where i⊂(0,T). At the beginning, we proposed an accurate method, which will not incur any meaningless waiting. Then, in consideration of the performance of the algorithm, we proposed a flexible version.

Firstly, we want to know every possible status of MDL, which status can help us to analyze the benefits from waiting one time slot. For better understanding, we make an example of this calculation. We assume there are two neighbors of the infected root node of DT, *A* and *B*. So, there are four possible statuses of *A* and *B*, like AB, $\bar{A}B$, $A\bar{B}$, $\bar{AB}$, where *A* is the event that one node get infected and $\bar{A}$ is not.

After analyzing the possibility of each status and their benefit, we can have the expected benefit from waiting one time slot. Now, in Algorithm 3, $V_{B}^{t=\theta }$ and $V_{B}^{t=\theta +1}$ are corresponding benefit sets to $V_{I_{\theta }}$ and $V_{I_{\theta +1}}$. The idea of Algorithm 3 is something like ‘*binary decision tree*’ as shown in line (5–7), by using these tools so that we can know the possibility of each event. Thus, we can use Algorithm 3 to analyze the expected benefit, and then, we can make a decision that put the nodes which have a bigger benefit at t=θ at t=θ, and others wait to next time slot.

It is easy to verify that Algorithm 3 can calculate out $V_{B}^{t=\theta }$ and $V_{B}^{t=\theta +1}$ but with a higher computation complexity($O(2^{n})$). For that reason, we need to propose a fast algorithm to make it more available for a larger network. Using Monte Carlo simulation method to get the vaccines set is a well-studied method [35]. In contrast to [35], we need not find all the vaccine sets but just simulate the propagation procedure in one time slot, which has so much less complexity than [35] dose. So we adopt the idea of Monte Carlo simulation method and use it to calculate out the possible benefit of $V_{B}^{t=\theta +1}$. At the same time, we both have $V_{B}^{t=\theta }$ and $V_{B}^{t=\theta +1}$, and these are all we need.

Finally, we can get the complete DWPV Algorithm 4. In Algorithm 4, we first find $V_{I_{0}}$ and get its corresponding benefit $V_{B}^{t=0}$, then we use the *Monte Carlo Process* to get a simulated result of $V_{I_{1}}$ to check out that if we obtain some nodes with a higher benefit than $V_{I_{0}}$. After that, we put those vaccines with higher benefit on G(V,E) in this round and others wait to next time slot. We will keep this process going until $k^{*}=0$. There is another thing that should be noticed: when we decide to put one vaccine down, there must be some neighbors of that position that have been infected or we let these vaccines wait. This little trick can bring some benefit in case some unexpected things to happen, such as we put one vaccine on the G(V,E) but its neighbors are hardly infected as G(V,E) is always changing.
**Algorithm 3** Analyzing the benefit of each time slot.**Input:** A dominator tree with infected root node, one vaccine, $P_{u,v}$, $V_{B}^{t=\theta }$ and $V_{B}^{t=\theta +1}$**Output:** Expected benefit with one vaccine in layer 1 $V_{B}^{t=\theta }$ and layer 2 $V_{B}^{t=\theta +1}$1:$P_{l1}\left[\;\right]$ {*Probability of transmit to layer 1 according to* $P_{u,v}$}2:**for** i=1 **to** $2^{N_{1}}$ **do**3:  Initialize $S_{all}=\left[i\right]$ with 1 $S_{benefit}=\left[i\right]$ with 0{*The set of all kinds of sort in the situation of layer 1 and The maximum benefit will owned when one node is selected*}4:**end for**5:**for** i=1 **to** $N_{1}$ **do**6:  Get all the immunization benefit from layer 1, namely, we get $V_{B}^{t=\theta }$7:**end for**8:**for** i=1 **to** $N_{1}$ **do**9:  Get all the nodes infection possibility in layer 2, which are conveyed from layer 110:**end for**11:**for** i=1 **to** $N_{2}$ **do**12:  Get all the immunization benefit from layer 2, namely, we get $V_{B}^{t=\theta +1}$13:**end for**14:**return** $\left[V_{B}^{t=\theta },V_{B}^{t=\theta +1}\right]$
**Algorithm 4** DWPV Algorithm.**Input:**
G(V,E), $I_{0}$ and *k***Output:**
$\varphi_{G,I_{0}}\left(V\right)$1:$k^{*}=k$2:**while** $I_{1}\neq$ NULL and $k^{*}>0$ **do**3:  G(V,E)→DT4:  $V_{I_{0}}=$ HeapSort(k, using Cal-Benefit() to calculate out every node’s benefit of $MDL_{I_{0}}$)5:  Using Monte Carlo Process to get $I_{1}$6:  $V_{I_{1}}=$ HeapSort(k, using Cal-Benefit() to calculate out every node’s benefit of $MDL_{I_{1}}$)7:  **for** i=0 **to** $k^{*}$ **do**8:      j=09:      **if** $\beta \left(V_{I_{0}}\right)\left[i\right]>\beta \left(V_{I_{1}}\right)\left[i\right]$ and neighborInfected(i) **then**10:            Removing node i from G(V,E)11:            j=j+112:      **end if**13:  **end for**14:  $k^{*}=k^{*}-j$15:  [G(V,E),I1]= IC-Process(G(V,E), $I_{0}$)16:**end while**17:$\varphi_{G,I_{0}}\left(V\right)=$ IC-Process(G(V,E))


### 4.5. IC Model to SIR Model

In the beginning of our paper, we introduced two classical propagation models which could represent the process of virus transmission. In all of our paper, we illustrate our method on IC model. However, the SIR model is also important to the virus controlling problem, and it will be our future work to make our method adapted to this model. Now, we will show how to exchange from IC model to SIR model. It is obvious that SIR has a very complicated process that we can not easily describe, so we want to follow the analyzing process of IC model and find a right place and time to put vaccines on G(V,E). The difference between those two models is on R. In IC model, we treat R is 1, which means each infected node just has one chance to infect its neighbors. In SIR model, $R\in \left[0,1\right]$, which means before the node is recovered, every infected node has a multi-chance to infect its neighbors. Here, we convert this multi-try infecting action into one-chance through calculating the entire possibility. Specially, the probability of being infected by one node is *P* and after *n* times trying (n→∞) could be illustrate like ${\left(1-R\right)}^{n}*{\left(1-p\right)}^{n-1}*P$. Once being infected, it will be added to the infected set. The relation between the infected node and its neighbors can be represented as

(5) $$\begin{array}{c} P_{SIR}=\left(1-R\right)\times P\left[\frac{1}{1-(1-R)\times (1-P)}\right], \end{array}$$

then we just need to use $P_{SIR}\left(u,v\right)$ to replace P(u,v), and it will make SIR model described as IC model.

## 5. Experiments

In this section, we will illustrate the experiment results of our algorithm. Moreover, we use the best performance strategy (DAVA [25]) of SVP problem to be the comparison algorithm. The final results have shown that we have a better performance as compared to DAVA.

### 5.1. Experiments Setup

#### 5.1.1. Real World Data Sets

**Figure 6:**
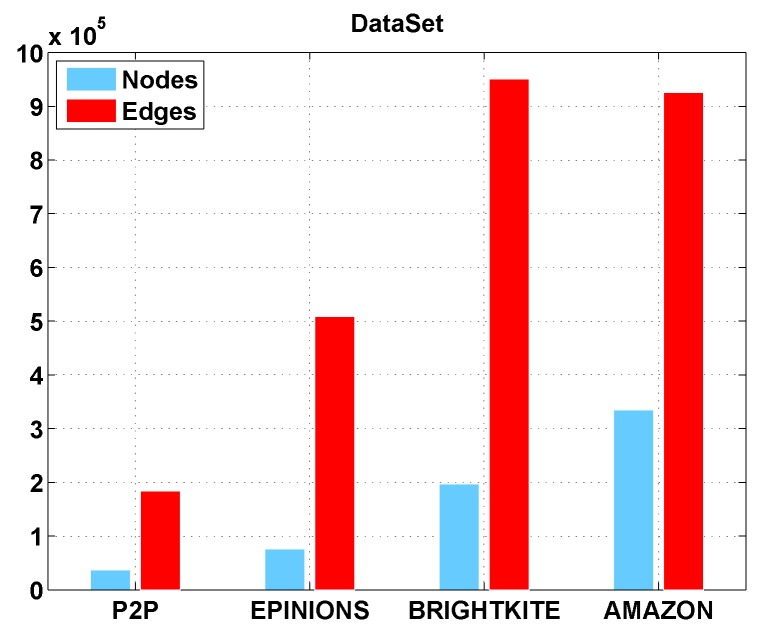
DataSet.

In our experiments, we run the algorithms on some real-world data sets [36] (as shown in Table 2 and the main attributes of these data sets have shown in Figure 6). Every set has a different topology so that we can test our method for different scenes. The normal and the large scale of a network are both used in our experiments for comprehensive performance testing. Real-world data can testify our strategy in a detailed way, which will be really helpful for designing a realistic algorithm [37,38].

The first set, P2P, a peer-to-peer network file shared by Gnutella, each node represents a host and each edge between nodes represents the connectivity of two hosts. We use it to simulate computer virus spreading and protecting. The second set, EPINION, collected by a Website and it is a who-trust-whom social network. Each person in this set has a relationship between other nodes and the relationship relies on whether you trust him/her. This trust-based model network is well studied in following years [39]. From that network, we can examine our algorithm in the scenario that a rumor breaks out in a network and how can we stop this rumor through our method. The third set, BRIGHTKITE, a location-based social network [40], edges represent the relationship between each node like EPINION is, but the number of nodes is much bigger than EPINION which has 197K nodes rather than 76K nodes in EPINION. The last set is from AMAZON, each node represents a product of the website and also has some relationships between itself and other products. We use this set because it has a large network size, which will help us decide whether our algorithm can be used in a network of this kind of size.

#### 5.1.2. Virus Transmission Probability

For the weight of each edge, we would like to set as 0.2, 0.4, 0.6 as these can stand the ordinary transmission probability that happens in our usual life. Besides, different transmission probabilities can reflect the performance of our algorithm in different scenes as the transmission probability will directly affect the propagation of the virus.

#### 5.1.3. Initial Infected Nodes Set

At the beginning of our experiments, we set the number of infected nodes as 100 and these nodes are selected at random. To better examine of our algorithm, we have changed the $|I_{0}|$ from 100 to 200 and 500 to see the difference that would be brought by $I_{0}$.

To get accurate results, we run our experiment 100 times to get an average result. All there experiments were conducted on a server which has an Intel Xeon E5-2660 4 cores CPU (2.20 GHz) and a 28 G RAM, the OS is 64-bit Ubuntu Server 14.04 LTS.

### 5.2. Results Analyzing

According to our extensive experiments, the results have shown that our method has a better benefit as compared to previous works. From those figures, we could clearly see that the performance of our algorithm is better than DAVA. When the virus transmission process is over, the last number of healthy nodes are far more than the DAVA algorithm has and DAVA has been proved as the best performance algorithm for saving nodes in graph.

#### 5.2.1. Different Data Set

**Figure 7:**
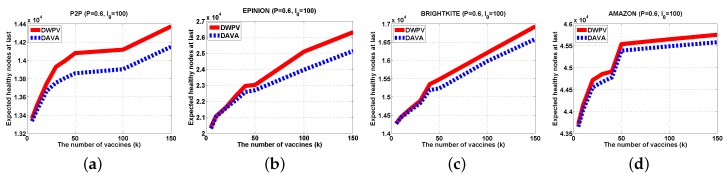
Experiments Results $\left(P=0.6,I_{0}=100\right)$. (**a**) P2P; (**b**) EPINION; (**c**) BRIGHTKITE; (**d**) AMAZON.

**Figure 8:**
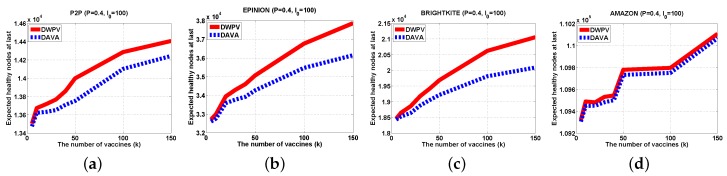
Experiments Results $\left(P=0.4,I_{0}=100\right)$. (**a**) P2P; (**b**) EPINION; (**c**) BRIGHTKITE; (**d**) AMAZON.

With a small data set, lowering the weight of each edge will not be of more benefit as compared to a big data set. The gap of DWPV between Figure 7a and Figure 8a is much smaller than the gap between Figure 7d and Figure 8d. For that reason, in a small data set, adding the number of vaccines will be a better choice. For some data sets in which nodes have a weak connectivity between other nodes like AMAZON, as shown in Figure 6, the effectiveness of putting vaccines is much less than other data sets. Under that circumstance, improving the defendable of each node will be a preferable choice. In a normal scenario, the DWPV always has more benefit than DAVA which proves the effectiveness of our solution. Especially, from Figure 7b and Figure 8b we can see that smaller weight of edges could bring more benefit than a bigger one.

#### 5.2.2. Different Number of Vaccines

**Figure 9:**
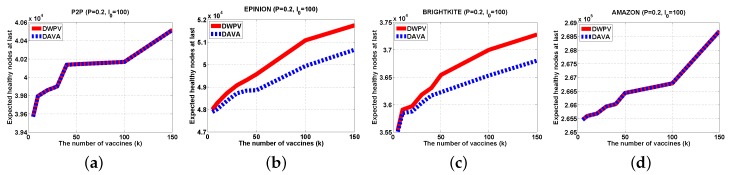
Experiments Results $\left(P=0.2,I_{0}=100\right)$. (**a**) P2P; (**b**) EPINION; (**c**) BRIGHTKITE; (**d**) AMAZON.

In Figure 7, we can see that DWPV has no obvious advantage. Through our cautious observation, then we investigate whether the gap between DWPV and DAVA is up to the utility of vaccines. Specially, as we increase the number of vaccines (from 5 to 150) in Figure 7b, $\varphi_{G,I_{0}}\left(V\right)$ with SVP strategy gets a 30% elevation, but in AMAZON, there is just a 5% elevation. Thus, we believe that the performance of DWPV has a closer relationship with the utility of the vaccine. This phenomenon can also be found in Figure 7a with 5% elevation and Figure 9a with 2% elevation. It is remarkable that in Figure 9a, the DAVA and DWPV have the same result for *k* vaccines. As we noticed in previous sections, that is the worst case of DWPV as it has the same result as DAVA. That is also to say, the effectiveness of our method is always better than DAVA.

#### 5.2.3. Different Initially Infected Node Set

**Figure 10:**
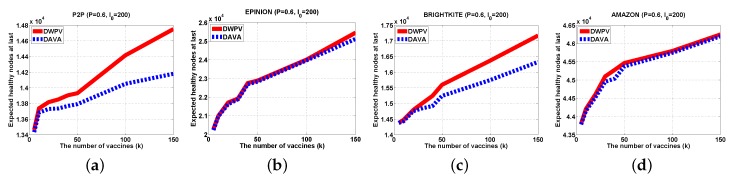
Experiments Results $\left(P=0.6,I_{0}=200\right)$. (**a**) P2P; (**b**) EPINION; (**c**) BRIGHTKITE; (**d**) AMAZON.

**Figure 11:**
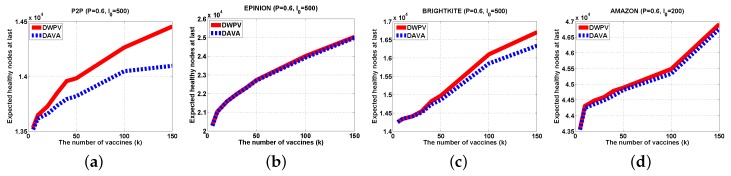
Experiments Results $\left(P=0.6,I_{0}=500\right)$. (**a**) P2P; (**b**) EPINION; (**c**) BRIGHTKITE; (**d**) AMAZON.

In order to verify the influence of difference $I_{0}$, we change the $I_{0}$ from 100 to 200 and 500 as shown in Figure 10 and Figure 11. There is no doubt that our method has a better performance again, however, there are some interesting things to focus on. Generally, the increasing number of initially infected nodes will lower the value of $\varphi_{G,I_{0}}\left(V\right)$, but in Figure 10b and Figure 11b we have not seen this happen. That is to say, adding the number of initial nodes will not always directly affect the $\varphi_{G,I_{0}}\left(V\right)$. The point is that the utility of vaccine may be reduced by the increasing number of initially infected nodes and this will lead to the decrease of the advantage of our method, like Figure 11b.

In conclusion, (1) the performance of our method is always better than DAVA; (2) lower down, the transmission probability will always get a higher $\varphi_{G,I_{0}}\left(V\right)$ but this not same lower than the number $I_{0}$.

## 6. Related Work

The problem of node immunization has been studied for a quite long time, most of the existing works try to allocate their vaccines before they know the initially infected nodes. Wang et al. [4,5] and Tong et al. [6,7] started their works on an arbitrary graph, while Madar et al. [41] studied this problem on complex networks, like power law graphs. Cohen et al. [42] and A. L. Buchsbaum et al. [43] analyzed the *acquaintance immunization* method that the vaccinated nodes have the most number of degrees in their network, for the SIS model and the SIR model. Hayashi et al. [3] described the propagation process by using SHIR (Susceptible, Hidden, Infectious, Recovered) model. Besides, Kimura et al. [44] introduce a novel solution to minimize the spread of contamination by blocking links rather than immunize nodes. However, none of above works based on the more realistic scenario that taking the initially infected nodes into the immunization strategy.

Some more practical works have been proposed by Zhang et al. [25] and its extending work [26], in which immunization decisions are based on the known initially infected nodes. They formalize the vaccines placement problem as Data-Aware Vaccination problem and convert the original graph into a dominator tree to distribute all vaccines on the neighbors of the root immediately. However, they miss a major concept in these algorithms that the benefit of putting the vaccine on one node is the dynamic change. Hence, using these strategies would sacrifice a great number of healthy nodes which could have been saved by a multi-step distribution.

While DWPV employs an dynamic-like vaccines placement strategy, it significantly differs from all past approaches [29,45,46] in how it defines the *dynamic*. Past schemes assume the network itself is changing, and their strategies are focusing on these changes. If these changes of the network are slight, they still choose to put all vaccines on the network at once. DWPV uses a simulation-based method to estimate how the limited vaccines should be distributed over the propagation process, and the vaccines are placed in batches on the appropriate nodes and at the appropriate time. Compared with previous well studied simulation-based methods [35], DWPV differentiates itself in the scope of simulation. DWPV simulates the transmission process just around the limited scope of these candidate nodes, and we need no more simulation once all vaccines have been placed, which reduces the computation complicity to a great extent and makes our algorithm more scalable than [35].

Finally, even if the strategy has been made by DWPV, we will put the vaccines on the network until a neighbor of the designated node has been infected, which gives our algorithm a further protection of the stochastic propagation model as compared with [25,26].

## 7. Conclusions

In this paper, we introduce a novel method to protect healthy nodes from be infected as best as we can with limited vaccines. In contrast to previous work, we first formulate our problem to a dynamic vaccine placement problem. Then, we showed that, even if the problem is the same, there is a significantly higher benefit with the same number vaccines (up to 30%). After that, we proposed an accurate solution to help us make a decision about when to put the vaccines on the graph by calculating the benefit of waiting one time slot or not waiting. Finally, for considering the calculation time restriction, we proposed a fast algorithm for our method, which is somewhat less accurate but is time efficient (computational is linear time). The extensive experiment shows our algorithms are much better than the previous works. 

## Figures and Tables

**Table 1:** List of notations.

Notation	Definition
G(V,E)	a propagation network, in this paper, we define our problem on undirected graph but not limited
$P_{u,v}$	the probability of *u* infected by *v*, and vice versa
SIR	transmission model with three status, *susceptible* (healthy), *infected* and *recovery*
*R*	recovery probability, one node from *infected* to *recovery* in SIR model
IC	a SIR-like model but that every nodes have only one chance to infect their neighbors
$I_{0}$	initial infectious nodes set
V	set of vaccines, which is used to stop or release the propagation of malicious things in G(V,E)
*k*	the budget of vaccines
$I_{i}$	infectious nodes set at time *i*
$\varphi_{G,I_{0}}\left(V\right)$	expected healthy nodes as the virus propagation process is over, and increasing its numerical value is the main target of this paper
β(n)	expected value of saving healthy nodes by vaccinating *n* in G(V,E).

**Table 2:** Data set for our experiment.

Data Sets	P2P	EPINION	BRIGHTKITE	AMAZON
#Node	63K	76K	197K	335K
#Edge	180K	509K	950K	926K
#Connected Component	12	2	547	1
MAX Component	62K	76K	57K	335K
